# Endogenous leukemia inhibitory factor protects photoreceptor cells against light-induced degeneration

**Published:** 2009-08-18

**Authors:** Sandra Bürgi, Marijana Samardzija, Christian Grimm

**Affiliations:** Laboratory for Retinal Cell Biology, Department of Ophthalmology, Zurich Center for Integrative Human Physiology (CIHP), University of Zurich, Zurich, Switzerland

## Abstract

**Purpose:**

Expression of leukemia inhibitory factor (LIF) by a subset of Müller glia cells has recently been implicated in an endogenous survival response to photoreceptor injury in a model of inherited retinal degeneration. To investigate whether such a LIF-controlled survival pathway might be commonly induced upon photoreceptor injury independently of the nature of the toxic stimulus, we analyzed the role of LIF during light-induced retinal degeneration.

**Methods:**

*Lif^+/–^* and *Lif^–/–^* mice were exposed to 15,000 lx of white light for 2 h. Retinal morphology and rhodopsin content were analyzed nine days after light exposure. Gene expression studies were done using real-time PCR. Protein levels were determined by western blotting using specific antibodies.

**Results:**

A lack of LIF reduced survival of photoreceptor cells after light exposure. In the absence of LIF several genes encoding molecules involved in the Janus kinase/signal transducer and activator of transcription (Jak/STAT) signaling pathway were not activated after light exposure. Presence or absence of LIF did not affect AKT (also known as protein kinase B, PKB) signaling and had only a mild effect on extracellular regulated kinase (ERK) phosphorylation. Stress-induced glial fibrillary acidic protein (GFAP) induction was minimal in the absence of LIF.

**Conclusions:**

Our results suggest that increased retinal expression of LIF is a general response to photoreceptor injury. Independent of the nature of the toxic insult (gene mutation, light), LIF may activate an endogenous rescue pathway that protects viable photoreceptor cells, leading to an increased photoreceptor survival in the stressed retina. This defense system may depend on the Jak/STAT pathway and may involve endothelin 2 (EDN2) but not (or only minimally) AKT and ERK1,2 signaling.

## Introduction

Degenerative diseases of the retina are widespread. In Europe and North America, for people over age 60, age-related macular degeneration (AMD) is the leading cause of blindness and severe visual impairment [1]. Currently, no effective therapies are available to sustain or improve vision in those with AMD or retinitis pigmentosa (RP). Several strategies are being followed to develop therapeutic approaches, one of which involves neuroprotection. However, in order for effective pharmaceuticals to be developed, the molecular events occurring in the degenerative retina need to be understood. Of special importance are mechanisms that are common to many, if not most, disease categories. Knowledge of these mechanisms may provide the basis for the inhibition of pro-apoptotic pathways or the activation of endogenous anti-apoptotic signaling systems.

It is well known that the retina can induce self-protecting mechanisms that help photoreceptors survive toxic stress situations. The concept of preconditioning, for example, uses subtoxic stress levels to provoke an endogenous protective response. In the retina, preconditioning has been applied successfully to protect neuronal cells from ischemic damage [2,3] and light-induced degeneration [4,5]. Depending on the preconditioning protocol, the retina may use different mechanisms for this protection. Whereas ischemic and hypoxic preconditioning may involve heat shock protein 27 (HSP27), erythropoietin, and other factors [4,6-8], preconditioning by light has been shown to involve activation of leukemia inhibitory factor receptor (LIFR) [5].

We have demonstrated that LIF, one of the main ligands for LIFR, is strongly upregulated in retinas exposed to excessive levels of white light [9]. Furthermore, we identified LIF to be the central molecule in a retinal response to a stress, which is induced by the expression of a mutant rhodopsin transgene in VPP mice [10], a model for autosomal dominant retinitis pigmentosa (adRP) [11]. In the VPP or light-stressed retina, LIF is produced by a subset of Müller glia cells and is required to support survival of photoreceptors. Lack of LIF strongly accelerates disease progression in the VPP mouse leading to a fast loss of photoreceptor cells [10]. Without LIF, an extensive endogenous signaling cascade culminating in the production of the growth and survival factor fibroblast growth factor-2 (FGF2) is not activated. Together with results obtained by the light-induced preconditioning paradigm, this suggests that LIF may orchestrate a common response to photoreceptor stress.

Here we tested whether LIF may also be required to reduce photoreceptor loss after extensive light exposure and whether the response to light stress involves molecular mechanisms similar to the signaling cascade identified in VPP mice. We show that the presence of LIF indeed reduced the severity of degeneration also in the light damage model and that this protection uses a similar signaling system as detected earlier in the inherited model of retinal degeneration. Thus, LIF-mediated survival signaling seems to be a general response used by the retina to counteract stress situations endangering survival of photoreceptors. Therapeutic stimulation of the LIF pathway may provide an attractive approach to prevent or delay photoreceptor degeneration in a broad range of degenerative diseases of the retina.

## Methods

### Mice and light exposure

Animals were treated in accordance with the regulations of the Veterinary Authority of Zurich and with the statement of “The Association for Research in Vision and Ophthalmology” for the use of animals in research. *Lif^+/–^* mice were a generous gift of Bettina Holtmann and Michael Sendtner (University of Wuerzburg, Wuerzburg, Germany). Mice were mated with 129S6/SvEvTac mice (Taconic, Hudson, NY) to generate LIF knockout animals on the light-sensitive *Rpe65_450Leu_* genetic background [12]. To generate *Lif^–/–^* mice, we bred *Lif^+/–^* males with *Lif^+/–^* females. Offspring were genotyped by PCR using DNA isolated from tail biopsies and specific primer pairs (Table 1). Presence of the wild-type allele (774 bp) and/or the knockout allele (500 bp) was determined by agaorse gel electrophoreses of the amplification products. Six-week-old *Lif^–/–^* and *Lif^+/–^* mice were used for the experiments. For light exposure, mice were dark-adapted overnight and their pupils were dilated with 1% Cyclogyl (Alcon, Cham, Switzerland) and 5% phenylephrine (Ciba Vision, Niederwangen, Switzerland) 45 min before exposure. Mice were exposed for 2 h to 15,000 lx of white fluorescent light. After exposure, mice were kept in darkness for 12 h before they were either killed or returned to normal cyclic light conditions (12 h at 60 lx; 12 h dark) for 9 days.

**Table 1:** Oligonucleotides

**Genotyping**		
**Gene, allele**	**Forward**	**Reverse**
*Lif, wt*	AAATGCCACCTGTGCCATACGC	CAACTTGGTCTTCTCTGTCCCG
*Lif, KO*	CTCTAAGCCTGAACTCTCTCATCC	GATTCGCAGCGCAGCGCATCGCCTT
**Real-time PCR**		
**Gene**	**Forward**	**Reverse**
*Edn2*	AGACCTCCTCCGAAAGCTG	CTGGCTGTAGCTGGCAAAG
*Ednrb*	ACCTACAAGTTGCTCGCAGAGG	AAAACCTATGGCTTCGGGGAC
*Gfap*	CCACCAAACTGGCTGATGTCTAC	TTCTCTCCAAATCCACACGAGC
*Fgf2*	TGTGTCTATCAAGGGAGTGTGTGC	ACCAACTGGAGTATTTCCGTGACCG
*Socs3*	GGAGACAGATGAGGCTGGTGA	GGACCTACTGACCGAGAGAT
*Stat3*	CAAAACCCTCAAGAGCCAAGG	TCACTCACAATGCTTCTCCGC
*Lif*	AATGCCACCTGTGCCATACG	CAACTTGGTCTTCTCTGTCCCG
*Clc*	GCATCAACTCCGCAGCTTAG	CTGAACGCCATAGCCAGGTCT

This table shows primers used for genotyping and real time PCR.

### Morphology

For light microscopy, mice were euthanized using CO_2_ followed by cervical dislocation at various time points as outlined in the text and figure legends. Eyes were fixed in 2.5% glutaraldehyde in 0.1 M cacodylate buffer (pH 7.3) at 4 °C overnight. For each eye, the superior and the inferior retina was prepared, washed twice in cacodylate buffer for 15 min each, incubated in osmium tetroxide for 1 h, dehydrated, and embedded in Epon 812. Next, 0.5 µm sections were prepared from the lower central retina and counterstained with methylene blue.

### Rhodopsin

The rhodopsin content was determined after 16 h of dark adaptation as described [13]. Briefly, all manipulations were conducted under dim red light. One retina from each individual animal was removed through a corneal slit and suspended in 1 ml ddH_2_O. After centrifugation (15,000x g, 3 min, 19 °C) the supernatant was discarded, and retinas were resuspended in 0.7 ml 1% hexadecyltrimethyl-ammoniumbromide (Fluka Chemie, Buchs, Switzerland) in ddH_2_O, homogenized with a polytron (20 s, 3,000 rpm) and centrifuged as above. The absorption at 500 nm of the resultant supernatant was measured in a spectrophotometer (Cary 50, Varian; Zug, Switzerland), using a plastic cuvette (path length, 1 cm). The sample was exposed to 20,000 lx of white light for 1 min to bleach all present rhodopsin, and the spectrophotometric measurements were repeated. The amount of rhodopsin present per retina was calculated using the following formula derived from the Lambert–Beer equation:

$$Rho=vol\;\times \;c=vol\;\times \;\Delta abs_{500}\;\times \;{\left(e\;\times \;l\times \;n\right)}^{-\text{1}}$$

where *vol*=0.0007 l, *e*=4.2 × 10^4^ cm^−1^M^−1^, *l*=1 cm, and n=1.

### RNA isolation, cDNA synthesis, and real-time PCR

Retinas were removed through a slit in the cornea and snap frozen in liquid nitrogen. Total retinal RNA was prepared using the RNeasy RNA isolation kit (Qiagen, Hilden, Germany) including a DNase treatment to digest residual genomic DNA. At least 1 µg of total RNA was used for reverse transcription using oligo(dT) and moloney murine leukemia virus (M-MLV) reverse transcriptase (Promega, Madison, WA). cDNAs from individual animals were amplified in duplicates with respective primer pairs (Table 1) in a LightCycler 480 instrument (Roche Diagnostics AG, Basel, Switzerland) using SYBR Green I Master Mix (Roche Diagnostics AG). mRNA levels were normalized to β-actin, and relative gene expression was calculated using the comparative threshold cycle method (Roche Light Cycler software, Roche Diagnostics, Basel, Switzerland). Relative values were calculated using a suitable calibrator sample.

### Western blotting

Retinas were homogenized by sonication in 100 mM Tris/HCl, pH 8.0, and analyzed for protein content using Bradford reagent. Standard SDS–PAGE (12%) and western blotting of 40 µg total retinal extracts were performed. Briefly, protein homogenates were separated on a denaturing polyacrylamide gel (12%) and blotted onto a nitrocellulose membrane (BioRad Laboratories, Reinach, Switzerland) using a semi-dry blotting system (BioRad Laboratories). After blocking with 5% non-fat dry milk (Bio-Rad, Munich, Germany) in TBST (Tris/HCl 10 mM, pH 8; 150 mM NaCl; 0.05% Tween-20) membranes were incubated with primary antibodies at 4 °C overnight followed by a 1 h incubation at RT with horseradish peroxidase-conjugated secondary antibodies. Immunoreactivity was visualized using the Western Lightning Chemiluminescence reagent (Perkin Elmer, Boston, MA).The following antibodies were used for immunodetection: anti-phospho-Jak2 (#44–426; Invitrogen, Basel, Switzerland), anti-Jak2 (#44–406; Invitrogen), anti-phospho-STAT1 (#9171; Cell Signaling, Danvers, MA), anti-STAT1 (#9172; Cell Signaling), anti-STAT3 (#9132; Cell Signaling), anti-phospho-STAT3_Tyr705_ (#9131; Cell Signaling), anti-glial fibrillary acidic protein (GFAP; G-3893; Sigma-Aldrich, Buchs, Switzerland), anti-β-actin (sc-1616; Santa Cruz, Santa Cruz, CA), anti-phospho-Akt_Tyr473_ (#9271; Cell Signaling), anti-Akt (#9272; Cell Signaling), anti-phospho- extracellular regulated kinase 1,2 (ERK1,2; #9101; Cell Signaling), and anti-ERK1,2 (# 9102; Cell Signaling).

## Results

### Light-induced photoreceptor degeneration

**Figure 1:**
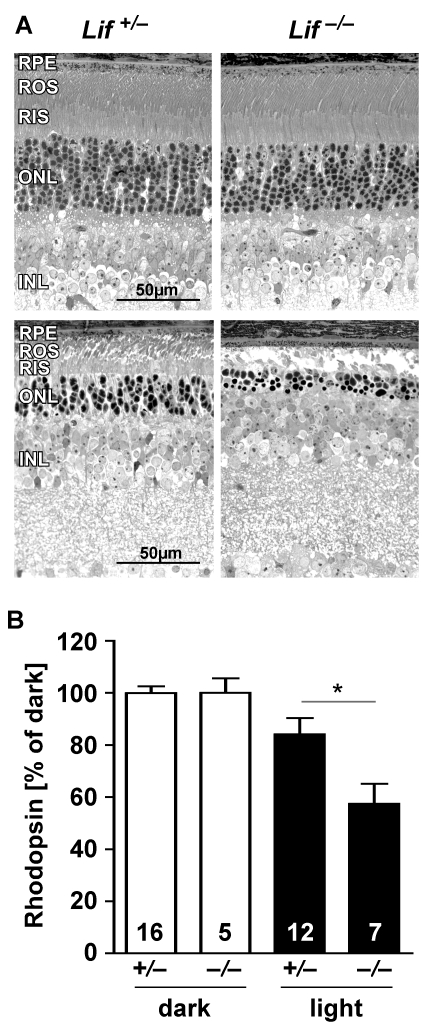
Lack of LIF increases light-induced photoreceptor degeneration. **A:** Retinal morphology of *Lif^+/–^* and *Lif^–/–^* mice was analyzed before (upper panels) or at 9 days after exposure to 15,000 lx of white light for 2 h (lower panels). Fewer photoreceptors survived light exposure in the lower central retina (the most affected region) in the absence of LIF in *Lif^–/–^* mice. Shown are representative sections of at least three animals. **B:** Rhodopsin levels were determined spectrophotometrically at 9 days after light exposure as a quantitative assessment of surviving rod photoreceptors in the whole retina. Rhodopsin levels after light exposure were expressed relatively to the respective dark controls, which were set to 100%. Note that values represent the average rhodopsin content of the whole retina, whereas the morphological pictures (**A**) show only the most affected areas. Abbreviations: retinal pigment epithelium (RPE); rod outer segments (ROS); rod inner segments (RIS); outer nuclear layer (ONL); inner nuclear layer (INL). Number of animals (*N*) is indicated for each group. The asterisk (*) indicates a p value of 0.0164 as calculated by a two-tailed *t*-test.

Retinal morphology of six-week-old *Lif^–/–^* mice was similar to wild-type (not shown) and *Lif^+/–^* mice (Figure 1A, upper panels). Retinal layers as well as photoreceptor cells were normally developed. Rod outer segments (ROS) and rod inner segments (RIS) had a regular structure with a normal thickness of the outer nuclear layer (ONL). In addition, dark-adapted levels of rhodopsin were similar in *Lif^+/−^* (450±45 pmol per retina, n=16) and *Lif^−/−^* mice (485±60 pmol per retina in, n=5), and intravitreal injections of recombinant LIF induced a similar molecular response in wild-type and *Lif^–/–^* retinas [10]. This suggests that LIF is not required for normal retinal development and that retinal cells lacking LIF are capable to induce molecular responses similar to wild-type cells.

Nine days after exposure to bright light, fewer photoreceptors survived in the lower central retinas of *Lif^–/–^* animals as compared to *Lif^+/–^* mice (Figure 1A, lower panels). To quantitatively assess the difference in the extent of light damage between the two genotypes, we measured rhodopsin levels, which are proportional to the amount of photoreceptors in the whole retina [14]. Nine days after light exposure, *Lif^–/–^* retinas had only 57% of the rhodopsin content of their undamaged dark controls, suggesting that 40% to 50% of photoreceptors had been lost as a consequence of light damage (Figure 1B). *Lif^+/–^* retinas, however, still had 84% of their normal rhodopsin levels, pointing to a photoreceptor loss of only 15% to 20% (Figure 1B). Note that rhodopsin measurements are averaging the rhodopsin (and thus photoreceptor) content in the whole retina, whereas morphologies (Figure 1A) focus on a small retinal region in the most affected retinal area.

### Lack of LIF disturbs signaling after light exposure

**Figure 2:**
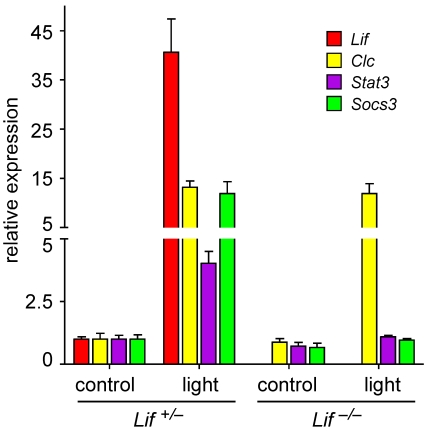
Lack of LIF prevents activation of STAT3 signaling. Relative levels of *Lif*, *Clc*, *Stat3*, and *Socs3* mRNAs (as indicated) were analyzed by real-time PCR in retinas of *Lif^+/–^* and *Lif^–/–^* mice before (controls) or at 12 h after exposure to 15,000 lx of white light for 2 h. RNA levels were expressed relative to levels in *Lif^+/–^* controls, which were set to 1. *β-actin* served as reference. Shown are means±SD of n=3.

**Figure 3:**
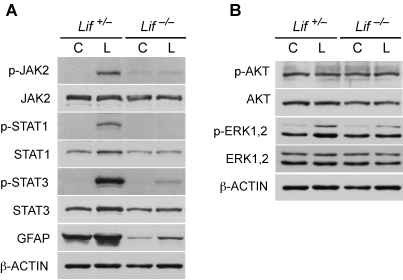
Lack of LIF alters the protein response pattern after light exposure. Levels of proteins (as indicated) were analyzed by western blotting in retinas of *Lif^+/–^* and *Lif^–/–^* mice before (controls, C) or at 12 h after exposure to 15,000 lx of white light for 2 h (L). Shown are representative blots of n=3.

We previously reported that light exposure activates not only expression of *Lif* but also expression of several other members of the Jak/STAT signaling pathway [9]. We therefore tested expression of *Lif* and cardiotrophin-like-cytokine (*Clc*), *Stat3*, and *Socs3* (suppressor of cytokine signaling) in control and light-exposed *Lif* heterozygous and *Lif* knockout animals. As in wild-type animals (data not shown) light exposure induced expression of all of these genes in *Lif^+/–^* mice. However, light-exposed *Lif^–/–^* mice upregulated expression of only *Clc* but not of *Stat3* or *Socs3* (Figure 2). Although CLC, which belongs to the interleukin-6 (IL-6) family of cytokines can also act as extracellular ligand to activate the Jak/STAT pathway [15], this suggests that LIF is essential for the normal retinal response to light stress. This is further supported by the different response of the *Lif^+/–^* and the *Lif^–/–^* retina on the protein level. Light exposure induced strong phosphorylation of JAK2, STAT1, and STAT3 in the *Lif^+/–^* but not in the *Lif^–/–^* retina. In addition, GFAP, a marker for Müller glia cell activity, was detectable at reduced levels even in unexposed *Lif^–/–^* control retinas, and its expression was not or only marginally induced by light in the absence of LIF (Figure 3A). In contrast, the absence of LIF did not affect the phosphorylation pattern of AKT and only marginally reduced the levels of phospho-ERK1,2 after light exposure (Figure 3B).

**Figure 4:**
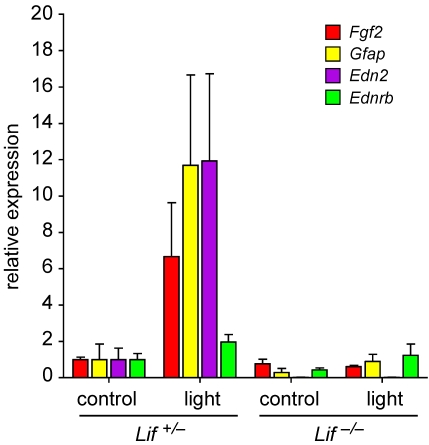
Lack of LIF alters gene expression after light exposure. Relative levels of mRNAs (as indicated) were analyzed by real-time PCR in retinas of *Lif^+/–^* and *Lif^–/–^* mice before (controls) or at 12 h after exposure to 15,000 lx of white light for 2 h. RNA levels were expressed relative to levels in *Lif^+/–^* controls, which were set to 1. *β-actin* served as reference gene for the relative quantification. Bars show mean values±SD (n=3).

In a model of autosomal dominant RP, we recently showed that LIF induces also endothelin 2 (EDN2) signaling, which leads to the expression of *Fgf2* and an increased survival of injured photoreceptors [10]. We thus investigated the same signaling system also in the model of light-induced retinal degeneration (Figure 4). Similar to the inherited model, photoreceptor injury induced expression of *Edn2*, *Fgf2*, and *Gfap* but had only a minimal effect on *Ednrb* expression. In the absence of LIF, light exposure did not induce any of these genes (Figure 4). The lack of GFAP activation is also reflected by the low levels of GFAP protein detected in the western blot (Figure 3).

## Discussion

Exposure of wild-type mice to excessive levels of white light strongly induces expression of *Lif* in a subset of Müller glia cells in the inner nuclear layer (INL) of the retina [9,10]. Here we analyzed the consequences of the absence of LIF in light-exposed animals and show that fewer photoreceptor cells survive exposure in *Lif^–/–^* mice. Jak/STAT and EDN2 signaling, which is normally induced after light stress, is lacking in the absence of LIF. As a consequence, expression of the growth and survival factor FGF2 is not induced and survival of photoreceptors is less sustained than in wild-type retinas.

It was recently shown that intravitreal application of recombinant LIF (rLIF) protects photoreceptor cells against light-induced degeneration [16]. Similarly, preconditioning by light provided protection against light damage through activation of LIFR [5]. Together with our data showing that lack of LIF increases the vulnerability of photoreceptors to light damage, this provides strong evidence for a potent neuroprotective role of LIF with a high capacity to support survival of photoreceptor cells. It is of significance that LIF acts as a neuroprotective not only in the light damage paradigm but also in models of inherited retinal degeneration. Photoreceptors expressing a mutant rhodopsin gene degenerate much faster in the absence of LIF [10]. This suggests that LIF controls a survival pathway that is generally induced upon a mild (light preconditioning) or strong (light damage, inherited degeneration) photoreceptor stress. Artificial interference with this pathway may provide a tool to protect photoreceptors and to prolong their survival in various disease pathologies. To do so, however, a detailed knowledge of the molecular mechanisms involved in LIF signaling is required. Here we show that the absence of LIF prevents expression as well as activation of members of the Jak/STAT signaling system (JAK2, STAT3, STAT1, SOCS3) in response to light stress. LIF seems to be the only cytokine that can induce the Jak/STAT pathway in the retina. CLC, which also belongs to the IL-6 family of cytokines is induced in the light-damaged retina but obviously does not have the capacity to activate JAK2 and its downstream signaling molecules in the light-exposed retina. In contrast to LIF, which signals through a bipartite receptor consisting of LIFR and glycoprotein 130 (gp130) [17], CLC requires the formation of a tripartite receptor including LIFR, gp130, and ciliary neurotrophic factor receptor (CNTFR) [15]. LIFR and gp130 are widely expressed in the retina including photoreceptors [16,18]. CNTFR, however, does not seem to be expressed in rodent photoreceptor cells [19] but has been found in ganglion cells and cells of the INL [19,20]. Since we isolated RNA and proteins from total retina and thus included cells of the INL and GCL, which express all three components of the CLC receptor, the absence of JAK2, STAT1, and STAT3 phosphorylation in LIF knockouts is surprising. Unlike other cytokines, however, CLC was reported to require the soluble receptor cytokine-like factor-1 (CLF-1) for the formation of an active complex to interact with the LIFR/gp130/CNTFR tripartite receptor [15,19]. It will therefore be of interest to analyze expression of CLF-1 in the physiologic and pathophysiological mouse retina to investigate the biologic significance of elevated CLC expression levels during light-induced retinal degeneration.

AKT has been associated with retinal neuroprotection in various situations [21,22]. However, in our light damage paradigm, we did not detect a differential regulation of AKT phosphorylation after light exposure [9]. Also, the absence of LIF did not influence the levels of p-AKT before or at 12 h after light offset (Figure 3). Similarly, phosphorylation of ERK1,2 was not affected or not strongly affected by the absence of LIF. The role for ERK1,2 in light-induced retinal degeneration is not clear. We noticed that light exposure induces phosphorylation of ERK1,2 similarly in retinas of susceptible and protected mice [9]. This makes it unlikely that ERK1,2 is actively involved in the degenerative process. However, it is possible that ERK1,2 is part of a protective pathway, independent of LIF signaling. Such a pathway, however, would not be able to protect photoreceptors from their increased vulnerability in the absence of LIF.

A central factor of the molecular response to photoreceptor injury seems to be EDN2. EDN2 is expressed by photoreceptors in the injured retina [23]. This expression, however, depends on LIF in a model of inherited retinal degeneration [10] as well as in the light damage model (Figure 4). Furthermore, activation of EDN2 receptor (EDNRb), which is expressed on Müller cells [23] by a synthetic agonist, increases resistance of photoreceptors against light stress [10]. Recently, Ueki and coworkers [16] demonstrated that injection of rLIF before light exposure is similarly protective. Since injection of rLIF induced expression of *Edn2* [10], it is likely, that this rLIF-mediated protection also involves EDN2 signaling, although this was not tested directly. Preconditioning by a subtoxic stimulus can induce a molecular response protecting the retina against a subsequent stronger insult [2,4,8]. Consequently, preconditioning by light was proven to be effective against light damage [5]. The molecular response induced by light-preconditioning involves activation of LIF expression and signaling through LIFR and STAT3 [5]. This is additional strong evidence for a central role of the LIF signaling system in retinal injury and photoreceptor protection.

Another striking observation is the strongly reduced expression of GFAP in mouse retinas lacking LIF (Figure 3). The reduced GFAP levels are not due to reduced numbers of Müller glia cells since *Lif^–/–^* mice have a similar spatial expression of glutamine synthase and comparable levels of cellular retinaldehyde binding protein (CRALBP) [10]. It has been reported, however, that development of astrocytes is impaired in brain tissue lacking LIF [24]. Since astrocytes enter the developing retina from the brain through migration along the optic nerve [25], it may be that the reduced GFAP levels in *Lif^–/–^* mice are a consequence of a reduced number of GFAP expressing astrocytes. Astrocytes are mainly found in the ganglion cell layer and around retinal blood vessels, where they may participate in the formation of the blood retina barrier (BRB) [25,26]. Thus, studies of the development and distribution of astrocytes in the wild-type and the *Lif^–/–^* mouse retina are warranted as well as investigations into the function of the BRB in these animals.

In summary, we show that lack of LIF signaling leads to increased photoreceptor death in the light-induced model (this work) and in a model of inherited retinal degeneration [10]. This suggests that the endogenous LIF system may be commonly activated in degenerating retinas, probably independently of the disease-causing stimulus. Targeting molecules of this signaling pathway by neuroprotective treatments may prove beneficial for the management of a large number of degenerative diseases.
